# Shifting Distribution and Lethality Profile of Suicide Methods in Türkiye Before, During, and After the COVID-19 Pandemic: A Nationwide Forensic Epidemiological Study

**DOI:** 10.3390/bs16101793

**Published:** 2026-10-01

**Authors:** Emrah Emiral, Gülsüm Öztürk Emiral

**Affiliations:** 1 Department of Forensic Medicine, Faculty of Medicine, Ankara University, Ankara 06620, Türkiye; 2 Department of Public Health, Faculty of Medicine, Ankara Yıldırım Beyazıt University, Ankara 06760, Türkiye; gulsum.ozturkemiral@aybu.edu.tr

**Keywords:** suicide, suicide method, lethality, COVID-19 pandemic, forensic epidemiology, Türkiye

## Abstract

Background/Objectives: The impact of the COVID-19 pandemic on suicide method distribution and lethality profiles remains insufficiently studied, particularly in the post-pandemic period. This study aimed to examine temporal changes in suicide methods and lethality profiles in Türkiye across three distinct periods: pre-pandemic (2016–2019), pandemic (2020–2022), and post-pandemic (2023–2025). Methods: National suicide statistics published by the Turkish Statistical Institute (TURKSTAT) for 2016–2025 were retrospectively analyzed. Suicide methods were classified as high-lethality (case fatality rate [CFR] > 50%) or low-lethality (CFR < 50%). Chi-square tests, Cramér’s V, and linear regression were applied. Methods without available CFR data (train/vehicle collision, self-immolation, other) were excluded from lethality analyses. Results: A total of 38,498 suicide deaths were recorded. The crude suicide rate increased from 4.03 to 5.36 per 100,000 (R^2^ = 0.909; *p* < 0.001), reaching its highest level in 2025. Firearm use declined during the pandemic (30.0% to 26.9%; *p* < 0.001), whereas jumping from height increased during the pandemic and remained elevated post-pandemic (12.1%, 13.9%, and 13.6%, respectively; *p* < 0.001). Chemical substance use temporarily increased during the pandemic (4.8% to 6.3%; *p* < 0.001), while natural/liquefied gas use increased post-pandemic (0.7%; *p* = 0.002). Conclusions: The crude suicide rate increased during the study period; however, this increase was not accompanied by a sustained shift toward higher-lethality methods. Post-pandemic suicide prevention strategies should be maintained, with particular attention to means restriction and sex-sensitive surveillance and prevention strategies.

## 1. Introduction

Suicide is a major global public health concern, responsible for approximately 700,000 deaths annually worldwide (Turecki & Brent, 2016; World Health Organization, 2025). During the COVID-19 pandemic, it was hypothesized that multiple factors, including quarantine measures, social isolation, economic hardship, health-related anxiety, and disruptions to mental health services, could influence suicide risk (Gunnell et al., 2020). Conversely, increased social solidarity, strengthened family support, and social protection policies implemented in some countries were proposed as potential protective factors. The net effect of the COVID-19 pandemic on suicide mortality therefore became an important area of research (da Cunha Varella et al., 2024; Pirkis et al., 2021). While global meta-analyses generally found no significant change in suicide rates during the pandemic (da Cunha Varella et al., 2024), elevated rates were reported in certain countries (Elsner et al., 2025). Evidence regarding the post-pandemic period remains limited, yet emerging data suggest that suicide mortality has risen again in some countries following the acute phase of the pandemic (Fouillet et al., 2023; Yang et al., 2025).

Türkiye reported its first confirmed COVID-19 case on 11 March 2020 and subsequently implemented targeted lockdowns and other containment measures that altered mobility, work, education, and social interaction. The economic consequences occurred against a background of pre-existing structural vulnerabilities. The OECD reported that employment and aggregate demand contracted markedly during the first pandemic wave, that tourism and hospitality were particularly affected, and that the pandemic exacerbated existing challenges related to unemployment, informal employment, indebtedness, inflation, and exchange-rate pressures (Organisation for Economic Co-Operation and Development, 2021). Evidence from Türkiye also indicates a substantial mental health burden during the early pandemic period. In a web-based cross-sectional study of 343 adults conducted in April 2020, 23.6% of participants scored above the cut-off for depression and 45.1% scored above the cut-off for anxiety. In multivariable analyses, female sex, urban residence, and a history of psychiatric illness were associated with anxiety; urban residence was associated with depression; and female sex, chronic disease, and a history of psychiatric illness were associated with greater health anxiety (Özdin & Bayrak Özdin, 2020). During the same period, psychosocial support hotline activities were implemented across all 81 provinces and provided support to approximately 236,000 people between April and December 2020 (World Health Organization, 2020). Taken together, the Turkish setting was characterized by simultaneous changes in daily routines and social interaction, economic uncertainty, mental health pressures, and an expansion of psychosocial support services. These contextual conditions provide the background for the theoretical perspectives considered below.

Several complementary theoretical perspectives may help contextualize how the pandemic could have affected suicide risk and method use. General strain theory identifies three major sources of strain: failure to achieve positively valued goals, removal or threatened removal of positively valued stimuli, and presentation or threatened presentation of negatively valued stimuli (Agnew, 1992). Although the theory was developed to explain crime and delinquency rather than suicidal behavior, its emphasis on adverse experiences and the negative affect they may generate provides a broader conceptual lens through which pandemic-related economic insecurity, social isolation, and disruption of valued roles may be considered as potential strains. In its original criminological formulation, the routine activity approach proposes that changes in everyday activities can alter the spatial and temporal circumstances and opportunities in which predatory criminal events occur (Cohen & Felson, 1979). Extending this perspective cautiously to suicidal behavior, pandemic-related changes in mobility, work, social interaction, and time spent in different settings may have altered the immediate availability of, or opportunity to use, particular suicide methods. This application represents a contextual hypothesis rather than a direct prediction of routine activity theory. The integrated motivational–volitional model provides a suicide-specific framework: it proposes that defeat and entrapment contribute to the emergence of suicidal ideation, whereas volitional moderators, including access to means, planning, exposure to suicidal behavior, and capability for suicide, influence the transition from ideation to suicidal behavior (O’Connor & Kirtley, 2018). Considered together, these perspectives suggest that the pandemic could have affected suicide risk without necessarily producing a uniform shift toward more lethal methods and that its effects may have varied according to sex, age, living circumstances, and access to means. These proposed mechanisms, however, cannot be directly tested using the aggregate data available in the present study.

In suicide epidemiology, monitoring death counts alone is insufficient; the distribution of methods used and their associated lethality levels are essential for a comprehensive understanding of suicidal behavior. In a meta-analysis of 34 studies, Cai et al. (2022) reported marked variation in case fatality rates (CFRs) across methods: firearms 89.7%, hanging 84.5%, drowning 80.4%, gas poisoning 56.6%, jumping from height 46.7%, drug poisoning 8.0%, and sharp instrument 4.0%. These differences underscore the importance of examining not only changes in suicide frequency but also potential shifts in method preference during crisis periods such as the COVID-19 pandemic. Beyond describing temporal trends, understanding changes in suicide method selection has important implications for suicide prevention. Monitoring shifts toward specific suicide methods and vulnerable demographic groups can support evidence-based prevention strategies, particularly means restriction interventions that have consistently demonstrated effectiveness across multiple settings (Steeg et al., 2025).

According to TURKSTAT data, the crude suicide rate in Türkiye increased from 4.03 per 100,000 in 2016 to 5.36 per 100,000 in 2025. The 2025 rate was the highest value recorded not only within the study period (2016–2025) but also in the broader national TURKSTAT series covering 2001–2025 (Turkish Statistical Institute, 2026). Existing studies on suicide in Türkiye have predominantly consisted of descriptive analyses focused on either the pre-pandemic or pandemic period (Battaloğlu İnanç, 2025; Emiral et al., 2022; İnce et al., 2026), while comprehensive comparative studies examining the pre-pandemic, pandemic, and post-pandemic periods together, particularly with respect to method distribution and lethality profiles, remain scarce. Understanding changes in method distribution is particularly relevant because method-specific surveillance provides information that can directly inform national suicide prevention policies and resource allocation (Nevarez-Flores et al., 2025).

This study aimed to examine changes in suicide rates and method distribution in Türkiye across three periods, pre-pandemic (2016–2019), pandemic (2020–2022), and post-pandemic (2023–2025), using national TURKSTAT statistics for 2016–2025, and to assess whether these changes differed by age group and sex.

## 2. Materials and Methods

### 2.1. Study Design

This was a retrospective forensic epidemiological study using an ecological time-trend design, based exclusively on publicly available, anonymized, aggregate national suicide statistics. No participant recruitment or interaction was undertaken, and the authors had no access to individual-level records, identifiable personal data, medical records, biological materials, or restricted microdata. Accordingly, ethical review and approval were not applicable to this study.

### 2.2. Data Sources

Data were obtained from publicly available TURKSTAT suicide statistics bulletins. TURKSTAT suicide statistics were based on records from the General Directorate of Security and the Gendarmerie General Command until 2012; from 2012 onward, the scope was expanded to include records from the TURKSTAT cause-of-death database, the Ministry of Justice General Directorate of Prisons and Detention Houses, and the General Staff of the Turkish Armed Forces. From 2018, records from the Coast Guard Command were additionally incorporated (Turkish Statistical Institute, n.d.). The entire study period (2016–2025) falls within this expanded scope. Data were drawn from three TURKSTAT bulletins: (1) suicides by method and sex, (2) suicides by method, sex, and age group, and (3) number of suicides and crude suicide rate. Only data for the years 2016–2025 were included in the analyses.

### 2.3. Study Periods

The study covered the calendar years 2016–2025. Period definitions are presented in Table 1.

### 2.4. Variables

The main study outcomes were the annual number of suicides, crude suicide rate per 100,000 population, suicide method distribution, and the proportion of high- and low-lethality methods. Suicide methods were examined in ten categories: (1) hanging, (2) chemical substances/drugs, (3) jumping from height, (4) drowning, (5) firearms, (6) self-immolation, (7) sharp instrument, (8) natural/liquefied gas, (9) jumping in front of train or vehicle, and (10) other. Demographic variables included sex (male/female) and age group (<15, 15–19, 20–24, 25–29, 30–34, 35–39, 40–44, 45–49, 50–54, 55–59, 60–64, 65–69, 70–74, 75+). For selected descriptive comparisons, adjacent age categories were combined into broader age groups (e.g., 15–34, 20–34, and 65+ years), as reported in the Results. As the study was based on aggregate national statistics, additional individual-level characteristics, including marital status, education, occupation, socioeconomic status, psychiatric history, and precipitating factors, were not available in the datasets used.

### 2.5. Lethality Classification

Suicide methods were classified according to case fatality rate (CFR) estimates reported in the meta-analysis by Cai et al. (2022). Methods with a CFR > 50% were classified as high lethality and included hanging (84.5%), firearms (89.7%), drowning (80.4%), and natural/liquefied gas (56.6%). Methods with a CFR < 50% were classified as low lethality and included chemical substances/drugs (8.0%), jumping from height (46.7%), and sharp instruments (4.0%). Cases recorded in the TURKSTAT categories “jumping in front of a train or vehicle,” “self-immolation,” and “other” were excluded from lethality analyses because corresponding CFR estimates were not available in the Cai et al. (2022) meta-analysis. These categories were retained for descriptive analyses of suicide method distribution.

### 2.6. Statistical Analysis

Categorical variables were analyzed using chi-square tests, with effect size estimated by Cramér’s V. Two separate simple linear regression models were used to assess temporal trends over the 2016–2025 period, with calendar year entered as the independent variable. Annual crude suicide rate was the dependent variable in the first model, and the annual proportion of high-lethality methods among cases with an available lethality classification was the dependent variable in the second model. Regression slopes, coefficients of determination (R^2^), and *p*-values for the slopes were reported. The general threshold for statistical significance was set at *p* < 0.05. For method-specific comparisons across the three study periods within the seven suicide methods included in the lethality analysis, a Bonferroni-corrected significance threshold of α = 0.007 (0.05/7) was applied. For comparisons of age distributions across the three study periods within the same seven suicide methods, a Bonferroni-corrected significance threshold of α = 0.007 (0.05/7) was also applied. For methods with a significant omnibus chi-square result, pairwise 2 × 2 chi-square tests were subsequently conducted to identify which study periods differed. The resulting *p*-values were adjusted for the three pairwise comparisons using the Bonferroni method. All analyses were performed using Python (SciPy v1.11).

## 3. Results

### 3.1. Overall Suicide Trends and Period Comparisons

A total of 38,498 suicide deaths were recorded in Türkiye between 2016 and 2025. Both the annual number of suicide deaths and the mean crude suicide rate increased across successive study periods, whereas males consistently accounted for approximately three-quarters of all suicide deaths. Detailed period-specific counts, annual means, crude rates, and sex distributions are presented in Table 2.

**Figure 1:**
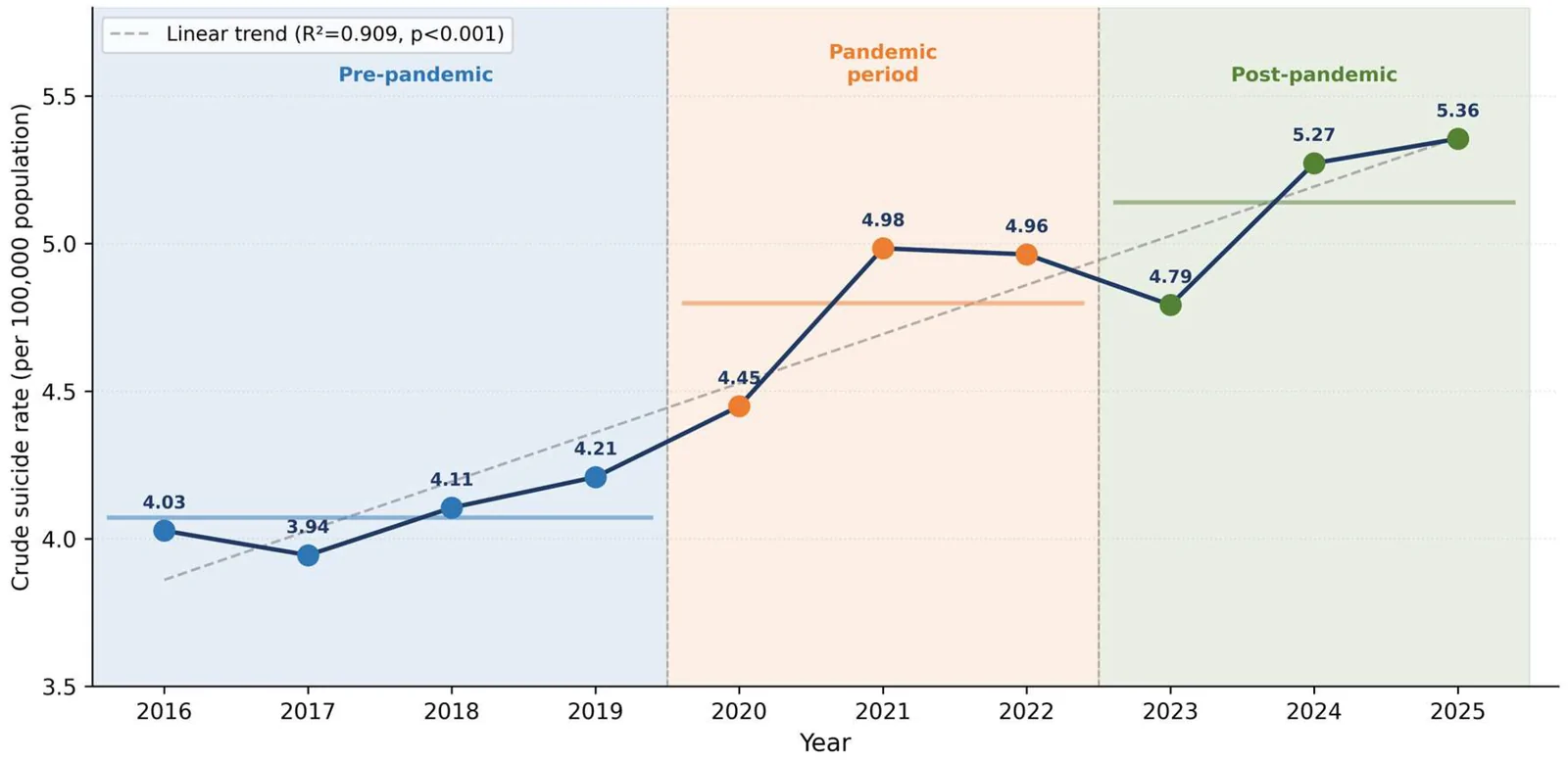
Crude suicide rate trend in Türkiye (2016–2025). Shaded backgrounds indicate study periods: blue = pre-pandemic (2016–2019), orange= pandemic period (2020–2022), and green = post-pandemic (2023–2025). Dashed vertical lines indicate period boundaries. Horizontal lines indicate period means.

Crude suicide rates showed an overall upward trend between 2016 and 2025 (Figure 1). Linear trend analysis demonstrated a significant mean annual increase of 0.17 per 100,000 population (R^2^ = 0.909; *p* < 0.001). Over the study period, the crude suicide rate increased by 33.0%, with the largest year-to-year increases observed between 2020 and 2021 (12.0%) and between 2023 and 2024 (10.0%). The rate reached its highest value in 2025, at 5.36 per 100,000 population.

### 3.2. Temporal Changes in Suicide Method Distribution

Among cases with available lethality classification, 12,485 (94.7%), 11,572 (95.5%), and 12,649 (95.8%) cases were included in the analyses for the pre-pandemic, pandemic, and post-pandemic periods, respectively. Hanging was the most frequently used method across all periods: 49.9%, 49.8%, and 49.1%. Hanging was followed by firearms (30.0%; 26.9%; 29.0%) and jumping from height (12.1%; 13.9%; 13.6%). The overall distribution of suicide methods differed significantly across periods (χ^2^ = 85.59; df = 12; *p* < 0.001; Cramér’s V = 0.034).

At the method level, firearm use declined during the pandemic (from 30.0% to 26.9%) and partially recovered post-pandemic (29.0%). Jumping from height increased during the pandemic (from 12.1% to 13.9%) and remained at a similar level post-pandemic (13.6%). Chemical substance use increased during the pandemic (from 4.8% to 6.3%) and returned toward pre-pandemic levels post-pandemic (4.9%). Natural/liquefied gas use increased in the post-pandemic period (0.7%). No significant changes were observed for hanging, drowning, or sharp instruments.

**Figure 2:**
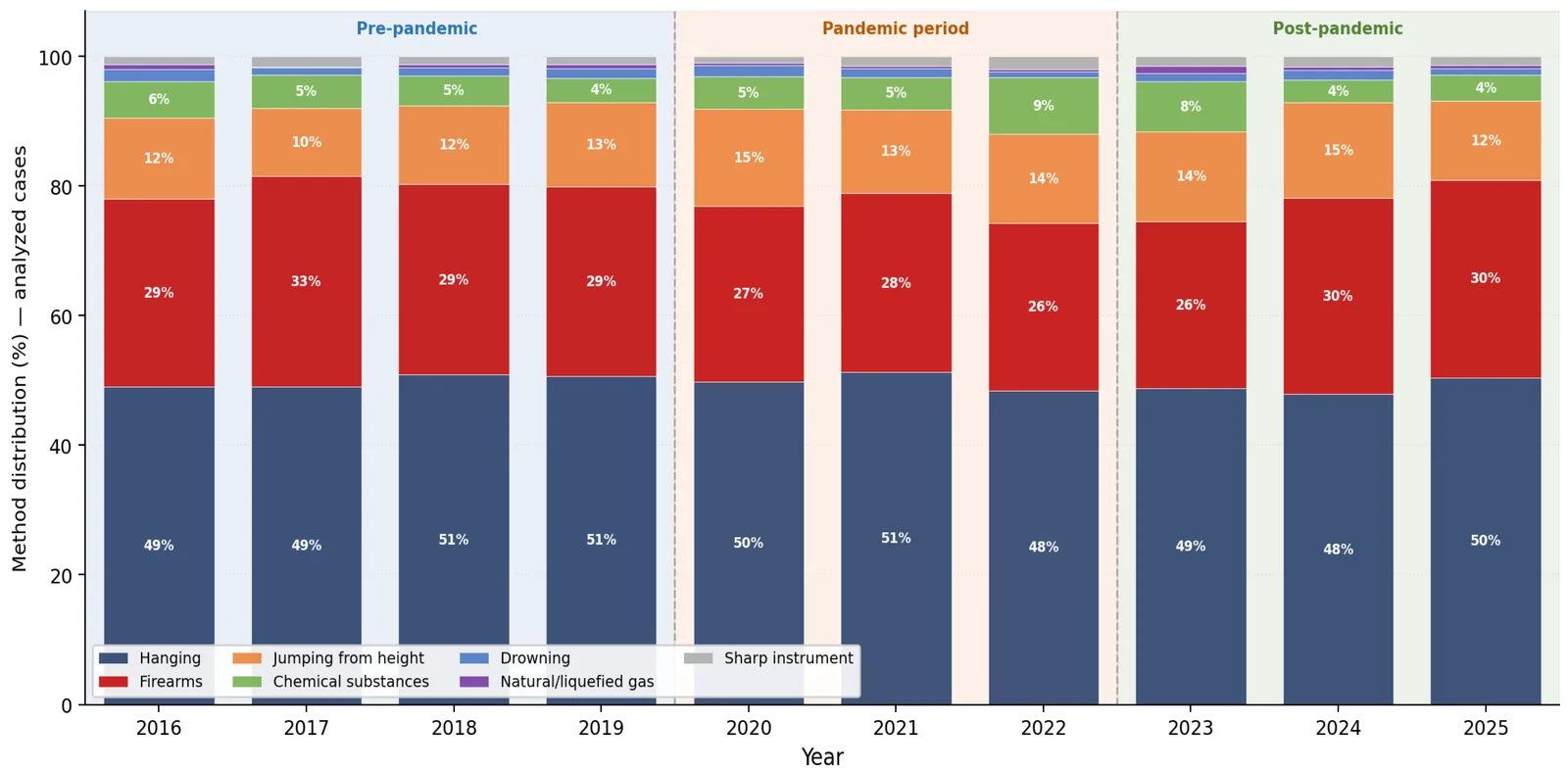
Annual distribution of suicide methods in Türkiye (2016–2025). The stacked area chart shows each suicide method in a different color. Dashed vertical lines indicate period boundaries. Methods without available CFR data (train/vehicle collision, self-immolation, and other) are excluded.

**Figure 3:**
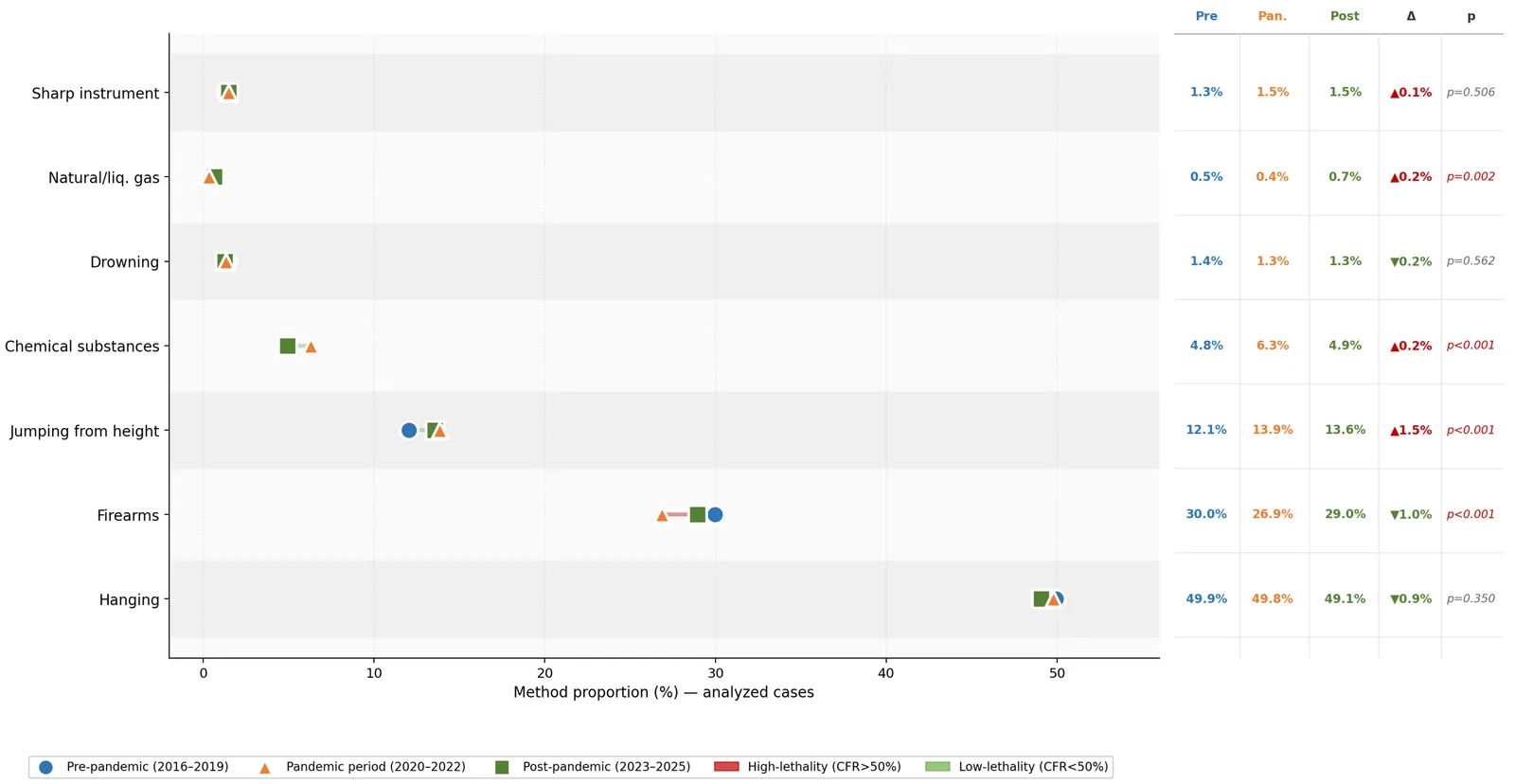
Period-specific changes in suicide method distribution. Circle: pre-pandemic (2016–2019); triangle: pandemic period (2020–2022); square: post-pandemic (2023–2025). Red: high-lethality methods; green: low-lethality methods. Upward/downward symbols indicate percentage change from pre-pandemic. *p*-values are based on chi-square tests with Bonferroni correction.

Pairwise post hoc comparisons confirmed that firearm and chemical substance use differed between the pandemic period and both the pre-pandemic and post-pandemic periods. For jumping from height, both the pandemic and post-pandemic periods differed significantly from the pre-pandemic period, whereas the pandemic and post-pandemic periods did not differ from each other. Natural/liquefied gas use differed significantly only between the pandemic and post-pandemic periods. The distribution of suicide methods by period and the corresponding statistical test results are presented in Table 3, while annual distributions and period-specific changes are shown in Figure 2 and Figure 3, respectively.

### 3.3. Lethality Profile Across Periods

**Figure 4:**
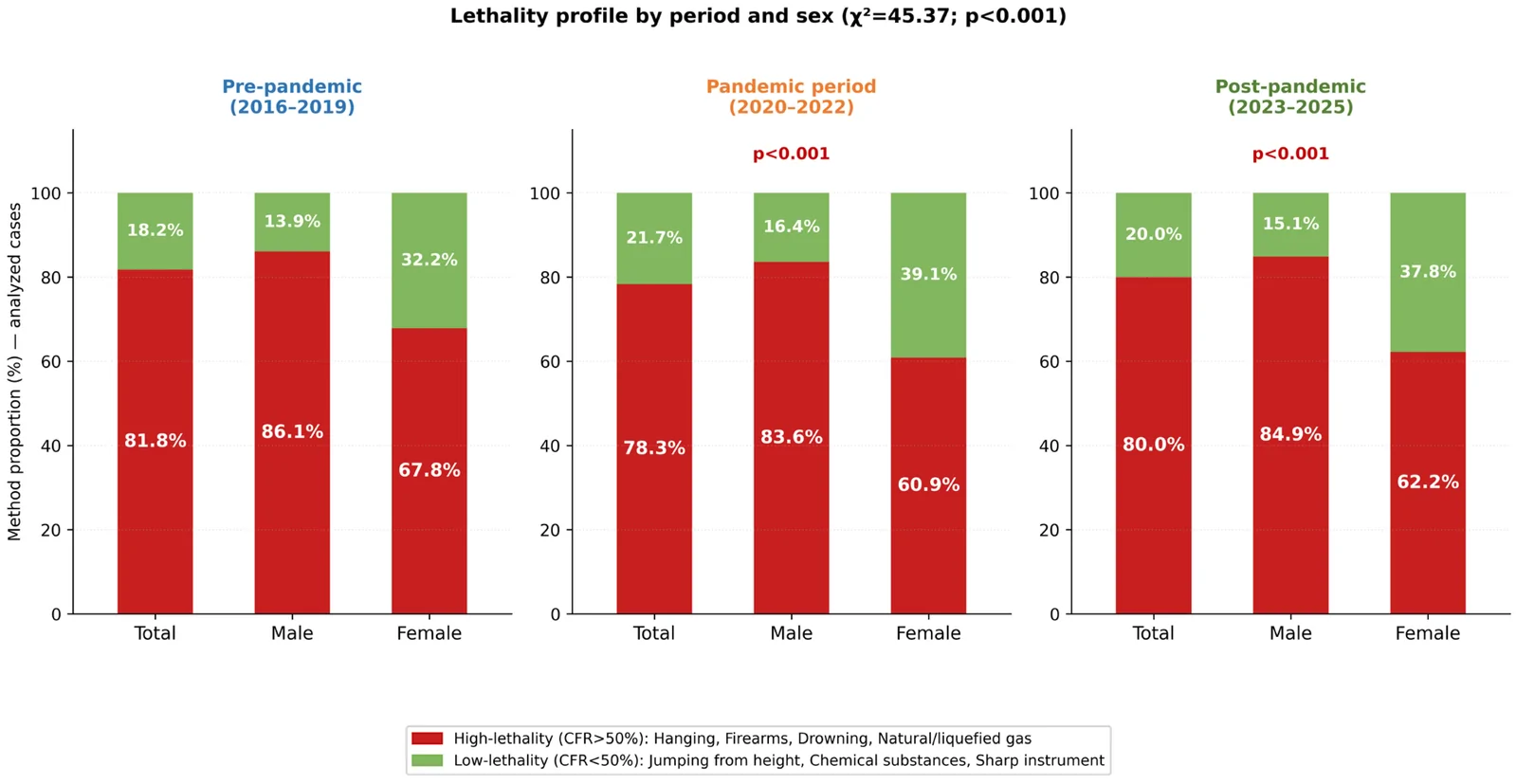
Lethality profile by period and sex. The proportions of high-lethality (red; CFR > 50%) and low-lethality (green; CFR < 50%) methods are shown among analyzed cases. Cases without CFR data were excluded. Classification was based on Cai et al. (2022). The overall distribution of high- and low-lethality methods differed significantly across study periods (χ^2^ = 45.37; df = 2; *p* < 0.001), and within each study period, the proportion of high-lethality methods was significantly higher among males than females (all *p* < 0.001).

Cases recorded in the TURKSTAT categories “jumping in front of a train or vehicle,” “self-immolation,” and “other” were excluded from lethality analyses (pre-pandemic: *n* = 694, 5.3%; pandemic: *n* = 550, 4.5%; post-pandemic: *n* = 548, 4.2%). Accordingly, 36,706 cases comprising 94.7–95.8% of total cases were included in lethality analyses. The proportion of high-lethality methods (hanging, firearms, drowning, and natural/liquefied gas) among analyzed cases was 81.8% (*n* = 10,214/12,485) in the pre-pandemic period, 78.3% (*n* = 9066/11,572) during the pandemic, and 80.0% (*n* = 10,117/12,649) post-pandemic. The proportion of high-lethality methods declined during the pandemic and partially recovered post-pandemic (χ^2^ = 45.37; df = 2; *p* < 0.001) (Figure 4). However, no significant linear trend in high-lethality method use was detected over the 2016–2025 period (slope: −0.27 percentage points/year; R^2^ = 0.113; *p* = 0.342).

### 3.4. Lethality Profile by Sex

When analyzed by sex, the proportion of high-lethality methods was 86.1%, 83.6%, and 84.9% in males, and 67.8%, 60.9%, and 62.2% in females, for the pre-pandemic, pandemic, and post-pandemic periods, respectively. High-lethality method use was consistently higher in males than females across all periods. In both sexes, the proportion of high-lethality methods declined during the pandemic and showed a partial increase post-pandemic. Within each study period, the proportion of high-lethality methods was significantly higher among males than females (all *p* < 0.001; Figure 4).

### 3.5. Suicide Method Distribution by Age Group

**Figure 5:**
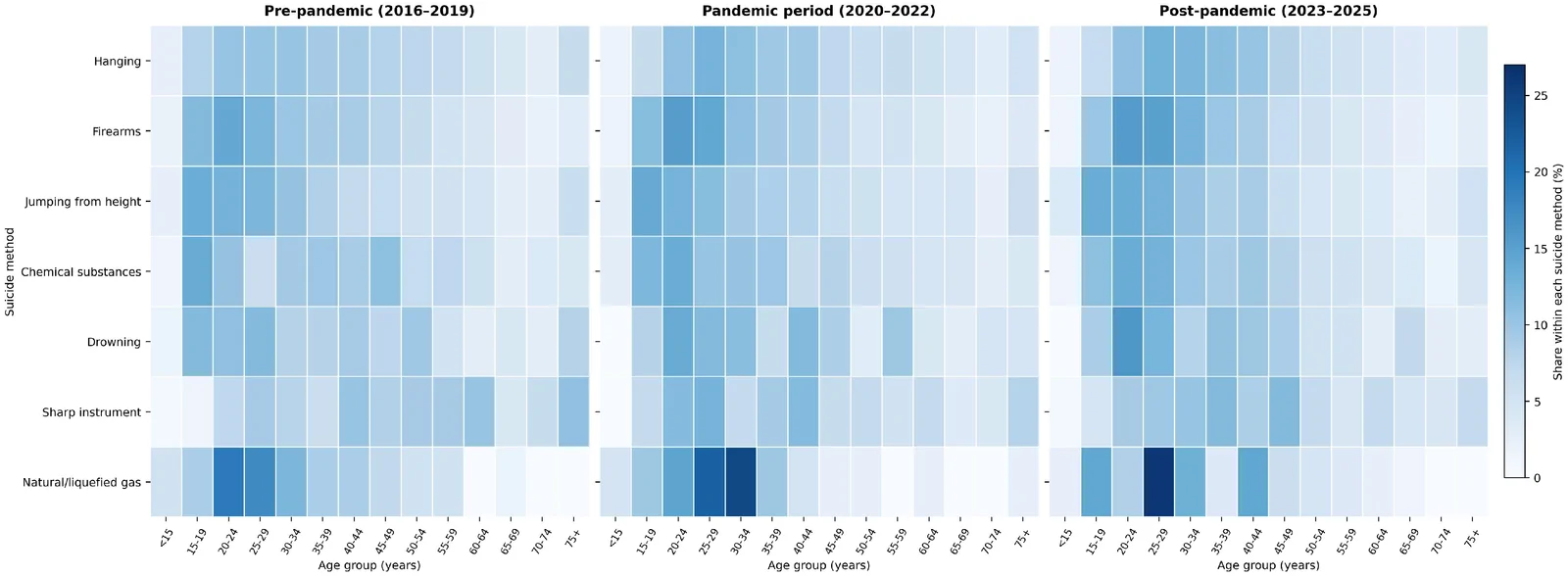
Age distribution within suicide methods across the three study periods. The heatmaps show the percentage distribution of age groups within each suicide method during the pre-pandemic, pandemic, and post-pandemic periods. Percentages were calculated separately for each method within each study period; therefore, each row sums to 100%. Darker shading indicates a greater proportional contribution of the corresponding age group.

The highest proportion of suicide deaths occurred in the 15–34 age group across all study periods (pre-pandemic: 42.6%; pandemic: 44.9%; post-pandemic: 46.5%). When the age distribution within each suicide method was examined, significant period-related differences after correction for multiple testing were observed for hanging and firearms. For hanging, the proportion of cases occurring among individuals aged 65 years and older decreased in the post-pandemic period (13.3%, 13.2%, and 10.6% across the pre-pandemic, pandemic, and post-pandemic periods, respectively; χ^2^ = 87.34; *p* < 0.001). The age distribution of firearm suicides also differed significantly across periods (χ^2^ = 32.34; *p* < 0.001), with the proportion of firearm cases occurring in the 15–34 age group increasing from 48.2% in the pre-pandemic period to 52.8% in the post-pandemic period. For chemical substance use, a descriptive increase was observed in the proportion of cases occurring among individuals aged 20–34 years, from 25.8% pre-pandemic to 34.0% during the pandemic and 36.3% post-pandemic; however, the overall age-distribution difference did not meet the Bonferroni-corrected significance threshold (χ^2^ = 16.12; *p* = 0.041). No statistically significant period-related differences in age distribution were observed for jumping from height, drowning, natural/liquefied gas, or sharp instruments after correction for multiple testing. Age-specific distributions of suicide methods across the three study periods are presented in Figure 5.

## 4. Discussion

### 4.1. Temporal Trends in Suicide Rates and Post-Pandemic Trajectories

This study evaluated changes in suicide mortality in Türkiye across the pre-pandemic, pandemic, and post-pandemic periods at the national level, and examined differences in suicide method distributions and lethality profiles. The crude suicide rate increased from 4.03 per 100,000 in 2016 to 5.36 per 100,000 in 2025, representing an approximately 33% increase over the study period. The finding that the mean crude suicide rate in the post-pandemic period (5.14 per 100,000) was higher than that during the pandemic period (4.80 per 100,000) indicates that suicide mortality did not return to pre-pandemic levels following the acute phase of the pandemic.

These findings are consistent with international studies reporting changes in suicide trends during and after the pandemic. A study using national death registry data in France observed a temporary decline in suicide mortality during COVID-19 restrictions, followed by a rebound after restrictions were lifted (Fouillet et al., 2023). Similarly, in Japan, an initial decline in suicide during the early pandemic months was followed by increases in subsequent periods (Tanaka & Okamoto, 2021). Findings from Germany demonstrated that suicide mortality exceeded expected levels outside of lockdown periods, a pattern interpreted by researchers as a “delayed effect” hypothesis (Elsner et al., 2025). The contrast between these country-specific patterns and the trajectory observed in Türkiye indicates that the pandemic was not associated with a uniform change in suicide mortality across countries and that pre-existing national trends and contextual conditions should also be considered.

The approximately 33% increase observed between 2016 and 2025 should therefore not be attributed solely to the COVID-19 pandemic. A recent joinpoint analysis of national mortality data from Türkiye identified a mild but non-significant estimated annual increase of 0.74% in the overall suicide rate between 2007 and 2019, followed by a significant and substantially steeper annual increase of 5.86% between 2019 and 2022. The same study found that the previously declining trend among females, corresponding to an estimated annual decrease of 2.27% between 2007 and 2019, reversed after 2019, with a significant annual increase of 8.11% between 2019 and 2022 (Durmuş et al., 2026). Our findings extend the observation period through 2025 and indicate that the upward trajectory was not confined to the acute pandemic period. Socioeconomic conditions may provide one possible contextual explanation. An econometric analysis using Turkish data from 1980 to 2019 found long-run associations between economic uncertainty, the Misery Index, defined as the sum of inflation and unemployment rates, and the number of suicides officially attributed to economic problems and business failures. In the same analysis, economic growth and industrial output were inversely associated with economically attributed suicides (Karasoy, 2026). Together, these studies provide a contextual basis for hypothesizing that pre-existing socioeconomic vulnerabilities may have interacted with pandemic-related disruption and its aftermath. However, this interaction was not directly tested, and neither these contextual associations nor the increase observed in our study establish causality at the individual level. Because the present aggregate dataset did not include employment, income, psychiatric diagnoses, family circumstances, or individual reasons for suicide, the relative contributions of economic strain, mental health burden, social isolation, service disruption, and other societal changes cannot be determined. The increase in Türkiye is therefore most appropriately interpreted as a cumulative population-level trajectory that may reflect several interacting pre-pandemic, pandemic, and post-pandemic factors rather than a direct effect of the pandemic alone.

### 4.2. Changes in Method Distribution: Comparison with International Findings

The persistence of hanging as the dominant method across all periods (~49%; *p* = 0.350) and the decline in firearm use during the pandemic (from 30.0% to 26.9%; *p* < 0.001) are among the notable findings of this study. The sustained predominance of hanging is consistent with multinational studies in the literature (Värnik et al., 2008; Wu et al., 2021). The high accessibility of hanging, the absence of specialized equipment requirements, and its relative insensitivity to sociocultural variation are among the factors proposed to explain its persistent dominance across countries and time periods (Värnik et al., 2008). These observations are further supported by findings from Frankfurt am Main, which detected no significant change in method distribution during the pandemic (Koelzer et al., 2024) and by a nationwide German study reporting no method shift between lockdown and non-lockdown periods (Elsner et al., 2025).

In contrast, firearm use declined during the pandemic and partially returned toward its pre-pandemic level in the post-pandemic period. In the integrated motivational–volitional model, access to the means of suicide is conceptualized as a volitional moderator that may influence the transition from suicidal ideation to suicidal behavior (O’Connor & Kirtley, 2018). In Türkiye, targeted lockdowns and other pandemic-related restrictions altered mobility, work patterns, social interaction, and daily routines (Organisation for Economic Co-Operation and Development, 2021). It is therefore plausible that these changes temporarily modified the circumstances in which suicidal acts occurred, including the immediate availability of, or opportunity to use, particular methods. The partial recovery in firearm use during the post-pandemic period may be compatible with such a temporary contextual influence; however, this interpretation remains hypothetical. The aggregate TURKSTAT data contain no information on firearm ownership, licensing, storage, place of occurrence, or the circumstances surrounding individual deaths. Consequently, it cannot be determined whether the observed decline reflected changes in firearm access, changes in the settings in which suicidal acts occurred, or other unmeasured factors. More broadly, international evidence indicates that greater firearm availability is associated with higher firearm suicide rates and that measures reducing firearm availability or restricting access among individuals at risk can reduce firearm suicides in some settings (Mann & Michel, 2016). These findings should nevertheless be extrapolated to Türkiye cautiously because firearm regulations, ownership patterns, and sociocultural conditions differ across countries.

The increase in jumping from height from 12.1% pre-pandemic to 13.6% post-pandemic (*p* < 0.001) is consistent with findings from some European studies reporting post-pandemic changes in suicide method selection (García-Ullán et al., 2022). Similarly, a Spanish study reported an increase in high-lethality suicide attempts during the post-pandemic period (García-Ullán et al., 2022). Although direct comparisons should be made cautiously because that study evaluated suicide attempts rather than completed suicides, these findings suggest that post-pandemic psychosocial conditions may have influenced method selection.

The increase in chemical substance use during the pandemic (6.3%) and its subsequent return toward pre-pandemic levels (4.9%; *p* < 0.001) may reflect changes in living conditions and accessibility of means during the pandemic period. However, the available data are insufficient to elucidate the mechanisms underlying this change. The post-pandemic increase in natural/liquefied gas use (0.7%; *p* = 0.002) represents a finding with limited precedent in the international literature and warrants further investigation in the Turkish context.

Taken together, although statistically significant differences were observed in method distribution, the very small effect size (Cramér’s V = 0.034) suggests that the COVID-19 pandemic did not fundamentally alter suicide methods in Türkiye, but led to limited changes in the relative frequency of certain methods. This finding is consistent with the international literature indicating that suicide methods vary across countries according to cultural characteristics, accessibility of means, and other structural factors (Nevarez-Flores et al., 2025; Värnik et al., 2008).

### 4.3. Lethality Profile Across Periods and Sex-Based Differences

In this study, the proportion of high-lethality methods declined during the pandemic and partially recovered in the post-pandemic period; however, no significant linear trend was observed over the 2016–2025 period. Notably, the proportion of high-lethality method use among females declined from 67.8% pre-pandemic to 60.9% during the pandemic, with only partial recovery in the post-pandemic period (62.2%).

The more pronounced decline in high-lethality method use among females may be interpreted cautiously in relation to possible changes in daily routines and access to means. In the integrated motivational–volitional model, access to means is conceptualized as a volitional moderator; however, these mechanisms were not directly examined in the present study (O’Connor & Kirtley, 2018). In a web-based cross-sectional study of 244 working women in Türkiye, 87.3% reported an increase in family workload and 83.0% reported an increase in workplace workload during the pandemic; depression, anxiety, and stress symptoms were also frequent in this sample (Küçükkaya & Kahyaoğlu Süt, 2022). These findings provide relevant context for the pandemic-related burdens experienced by some working women in Türkiye, but they neither establish an association with suicide method choice nor represent all women in the country. Accordingly, the observed decrease in the proportion of high-lethality methods among females may reflect changes in the relative distribution of methods, differential access to lethal means, or broader compositional changes among suicide deaths. These possible mechanisms should be investigated using individual-level data.

This finding is consistent with international studies reporting changes in female suicide patterns and method preferences during the pandemic. Studies from Japan reported marked increases in female suicide during the later pandemic period, associated with social isolation, economic vulnerability, and increased caregiving burden (Tanaka & Okamoto, 2021). Similarly, data from Spain indicated that while females used lower-lethality methods more frequently than males, high-lethality suicide attempts increased in the post-pandemic period (García-Ullán et al., 2022).

In the systematic review and meta-analysis by Cai et al. (2022), fatality rates were reported to be largely similar between males and females using the same method. Accordingly, sex differences in suicide mortality are thought to arise not merely from method lethality, but also from differences in method distribution. The “gender paradox in suicide”, in which completed suicide rates are higher in males despite higher rates of attempts in females, has been partly attributed to sex differences in method selection, with males more often using higher-lethality methods (Canetto & Sakinofsky, 1998). Multinational analyses from European countries have similarly demonstrated that males more frequently employ high-lethality methods such as hanging and firearms, while females tend to use lower-lethality methods (Värnik et al., 2008). The finding that males used high-lethality methods at consistently higher rates than females across all periods in our study is in line with this international literature.

Furthermore, the absence of a significant long-term linear trend in high-lethality method use (*p* = 0.342), despite partial recovery in the post-pandemic period, indicates that the increase in the crude suicide rate was not accompanied by a sustained shift toward higher-lethality methods. This finding highlights the importance of investigating the potential roles of mental health conditions, economic circumstances, and other social determinants in explaining the observed increase in suicide mortality. Given the very small overall effect size, these findings alone should not be interpreted as supporting resource-intensive or pandemic-specific changes to suicide prevention policy. Rather, they may inform the continued use of proportionate, context-appropriate surveillance and prevention measures beyond emergency periods.

Our findings have several implications for suicide prevention in Türkiye. International evidence and WHO guidance identify restriction of access to commonly used lethal methods as an important population-level suicide prevention approach, while emphasizing that interventions should be selected according to method prevalence, feasibility, available resources, and the local sociocultural context (Nevarez-Flores et al., 2025; World Health Organization, 2021). However, hanging remained the predominant method throughout the study period, and both Nevarez-Flores et al. and WHO acknowledge the limited availability of effective measures for restricting hanging outside institutional settings. In institutional settings, reducing access to ligatures and modifying potential ligature points may be appropriate, but community-level prevention in Türkiye cannot rely on means restriction alone. It should also include improved help-seeking, early identification of suicide risk, continuity of mental health care, and rapid access to crisis services (Nevarez-Flores et al., 2025; World Health Organization, 2021). Türkiye regulates the purchase, possession, and carrying of firearms through national legislation, principally Law No. 6136 and Law No. 2521 (Republic of Türkiye, 1953, 1981); however, to our knowledge, no national study has directly evaluated whether these regulations have reduced firearm-related suicide. Given the high lethality of firearms, secure storage, enforcement of existing licensing and registration requirements, and temporary restriction of access during periods of acute suicide risk may nevertheless be considered as context-appropriate preventive measures. The sustained increase in jumping from height supports the identification of recurrent locations and, where appropriate, the installation of physical barriers, fencing, safety nets, crisis-contact signage, and other site-specific interventions (Nevarez-Flores et al., 2025). Because the TURKSTAT category of “chemical substances” does not identify the agents involved, more detailed poison surveillance would first be required to determine whether agent-specific measures involving packaging, dispensing, safe storage, sales restrictions, or regulation are warranted. These method-specific interventions should be accompanied by systematic suicide risk assessment and rapid referral pathways in primary care, emergency departments, and community mental health services, with particular attention to population groups showing higher or changing suicide burden, including younger age groups and females, among whom changes in method-specific or lethality patterns were observed. During the pandemic, psychosocial support hotline activities were implemented across all 81 provinces of Türkiye and provided support to approximately 236,000 people between April and December 2020 (World Health Organization, 2020). Maintaining such capacity beyond acute emergencies and integrating it with crisis assessment, referral, and follow-up services may strengthen national suicide prevention efforts. Continued monitoring of method-, age-, and sex-specific patterns in TURKSTAT data would also allow prevention strategies to be adapted as population-level patterns change.

### 4.4. Strengths and Limitations

This study has several notable strengths, including its evaluation of the pre-pandemic, pandemic, and post-pandemic periods together; reliance on national-level TURKSTAT data; and systematic examination of changes in suicide method distributions and lethality profiles. Most existing studies examining the relationship between COVID-19 and suicide in Türkiye have focused on specific age groups, limited time frames, or local samples (Akın & Konar, 2025; Şimşek & Demircan, 2025) and comparative studies at the national level across these three distinct periods remain scarce. This study therefore contributes to a more comprehensive understanding of suicide epidemiology in Türkiye in the context of the pandemic. The use of a 10-year time series further allowed for the assessment of long-term trends.

Several limitations should be acknowledged. Because no individual-level data were available, true case fatality rates could not be calculated. Lethality classification was therefore based on published method-specific CFR estimates reported by Cai et al. (2022). Cases in the jumping in front of a train or vehicle, self-immolation, and other categories were excluded from lethality analyses due to the unavailability of reliable CFR data. The possibility of incomplete registration or misclassification in TURKSTAT data cannot be entirely excluded. Additional individual-level demographic, clinical, and social characteristics, including marital status, education, occupation, socioeconomic status, psychiatric diagnoses, alcohol/substance use, and precipitating factors, were not available in the aggregate datasets used and therefore could not be assessed.

### 4.5. Future Research

Future research should investigate the factors underlying the approximately 33% increase in the crude suicide rate observed between 2016 and 2025. Studies covering longer pre- and post-pandemic periods and applying interrupted time-series or joinpoint methods may help distinguish pandemic-related changes from pre-existing secular trends and subsequent socioeconomic developments. Analyses using regional data could further examine whether changes in suicide rates and methods varied according to local unemployment, income, urbanization, mental health service availability, and other contextual characteristics. Where permitted, linkage of mortality records with forensic, health care, psychiatric, prescription, toxicological, law-enforcement, and socioeconomic data would allow individual-level assessment of psychiatric diagnoses, previous self-harm, substance use, employment, precipitating events, and access to suicide means. Such studies should examine whether the observed changes differed by sex and age and should specifically investigate the temporary decline in firearm use, the sustained increase in jumping from height, and the changing contribution of chemical substances among younger adults. Psychological autopsy and other qualitative studies may complement registry-based analyses by clarifying how pandemic-related stressors, social isolation, economic adversity, and changes in daily routines influenced suicidal behavior and method selection. Finally, studies incorporating both fatal and non-fatal suicide attempts are needed to generate Türkiye-specific estimates of method lethality rather than relying exclusively on case fatality rates derived from international evidence.

## 5. Conclusions

This study demonstrated that the crude suicide rate in Türkiye increased from 4.03 to 5.36 per 100,000 between 2016 and 2025, with the post-pandemic period exhibiting a higher suicide burden than the pandemic period itself. Among suicide methods, hanging remained the most frequently used throughout all periods. Firearm use declined during the pandemic and partially recovered post-pandemic, whereas jumping from height increased during the pandemic and remained elevated thereafter. Chemical substance use showed a temporary pandemic-period increase, while natural/liquefied gas use was highest in the post-pandemic period. In the lethality analysis, the proportion of high-lethality methods was 81.8% pre-pandemic, declined to 78.3% during the pandemic, and recovered to 80.0% post-pandemic; however, no significant long-term linear trend was observed. This finding indicates that the increase in the crude suicide rate was not accompanied by a sustained shift toward higher-lethality methods. Additionally, the decline in high-lethality method use observed among females during the pandemic had not fully returned to pre-pandemic levels by the post-pandemic period.

These findings highlight the importance of sustaining suicide prevention strategies beyond the pandemic period. Particular attention should be directed toward implementing context-appropriate means restriction interventions, strengthening accessible mental health and crisis-support services, and monitoring method-, age-, and sex-specific patterns. Integrating such surveillance into national suicide prevention programs may help identify emerging population-level changes and guide strategies tailored to Türkiye’s needs.

## Figures and Tables

**Table 1:** Definition of Study Periods.

Period	Years	Duration	Definition
Pre-pandemic	2016–2019	4 years	Reference period
Pandemic period	2020–2022	3 years	Calendar years corresponding to the main COVID-19 pandemic period
Post-pandemic	2023–2025	3 years	Period following the lifting of major public health measures

**Table 2:** Suicide Deaths, Annual Crude Suicide Rates, and Sex Distribution by Study Period in Türkiye, 2016–2025.

Period	Total *n*	Annual Mean (Range)	Mean Crude Rate (Range)	Male *n* (%)	Female *n* (%)
Pre-pandemic (2016–2019)	13,179	3295 (3168–3476)	4.07 (3.94–4.21)	10,084 (76.5%)	3095 (23.5%)
Pandemic period (2020–2022)	12,122	4041 (3710–4218)	4.80 (4.45–4.98)	9281 (76.6%)	2841 (23.4%)
Post-pandemic (2023–2025)	13,197	4399 (4089–4599)	5.14 (4.79–5.36)	10,300 (78.0%)	2897 (22.0%)

Crude rate: per 100,000 population.

**Table 3:** Distribution of Suicide Methods by Period and Chi-Square Test Results.

Method	Lethality Class *	Pre-Pandemic *n* (%)	Pandemic *n* (%)	Post-Pandemic *n* (%)	χ^2^	*p*
Hanging	H	6236 (49.9%)	5764 (49.8%)	6210 (49.1%)	2.10	0.350
Firearms	H	3743 (30.0%) ^a^	3108 (26.9%) ^b^	3662 (29.0%) ^a^	29.55	**<0.001**
Jumping from height	L †	1506 (12.1%) ^a^	1603 (13.9%) ^b^	1719 (13.6%) ^b^	20.07	**<0.001**
Chemical substances	L	598 (4.8%) ^a^	730 (6.3%) ^b^	625 (4.9%) ^a^	33.01	**<0.001**
Drowning	H	178 (1.4%)	153 (1.3%)	161 (1.3%)	1.15	0.562
Sharp instrument	L	167 (1.3%)	173 (1.5%)	188 (1.5%)	1.36	0.506
Natural/liquefied gas	H	57 (0.5%) ^ab^	41 (0.4%) ^a^	84 (0.7%) ^b^	12.35	**0.002**
Total	—	12,485 (100%)	11,572 (100%)	12,649 (100%)	85.59	<0.001

* H = high lethality (CFR > 50%); L = low lethality (CFR < 50%); classification was based on Cai et al. (2022). † Jumping from height had a CFR of 46.7% and was therefore classified as a low-lethality method. Jumping in front of a train or vehicle, self-immolation, and other categories were excluded from the lethality analyses because CFR data were unavailable. Bold *p*-values indicate statistical significance after correction for the seven method-specific comparisons (Bonferroni-corrected α = 0.007). Within each row, proportions that do not share the same superscript letter differ significantly in Bonferroni-adjusted pairwise post hoc comparisons.

## Data Availability

The data analyzed in this study are publicly available through the Turkish Statistical Institute (TURKSTAT) data portal at https://veriportali.tuik.gov.tr/en/press/58233, accessed on 7 July 2026. No restricted or proprietary data were used.
